# Financial Toxicity During Active Treatment for Head and Neck Cancer: Clinical Burden, Structural Determinants, and Supportive Care Perspectives

**DOI:** 10.3390/cancers18142248

**Published:** 2026-07-14

**Authors:** Ana Carolina Prado-Ribeiro, Luciana Estevam Simonato, Manoela Carrera, Thaís Bianca Brandão, Manoela Domingues Martins, Thomas P. Sollecito

**Affiliations:** 1 Instituto do Cancer do Estado de São Paulo (ICESP), University of São Paulo, São Paulo 01246-000, SP, Brazil; thais.brandao@hc.fm.usp.br; 2 Piracicaba Dental School, State University of Campinas (UNICAMP), Piracicaba 13414-903, SP, Brazil; manoela.martins@ufrgs.br; 3 National Institute of Science and Technology in Translational Biophotonics in Dentistry (INCT-BIOFOTO BUCAL), Porto Alegre 90010-150, RS, Brazil; luciana.simonato@ub.edu.br; 4 Bioengineering Graduate Program, Scientific and Technological Institute, Brazil University, São Paulo 08230-030, SP, Brazil; 5 Environmental Sciences Graduate Program, Institute of Science and Technology, Brazil University, Fernandópolis 15600-000, SP, Brazil; 6 Department of Life Sciences, State University of Bahia, Salvador 41150-000, BA, Brazil; manoelap@ufba.br; 7 School of Dentistry, Federal University of Bahia, Salvador 40100-150, BA, Brazil; 8 Department of Oral Pathology, School of Dentistry, Federal University of Rio Grande do Sul, Porto Alegre 90010-150, RS, Brazil; 9 Department of Oral Medicine, Penn Dental Medicine, University of Pennsylvania, Philadelphia, PA 19104, USA; tps@upenn.edu

**Keywords:** head and neck cancer, financial toxicity, quality of life, supportive oncology, treatment adherence

## Abstract

Background/Objectives: Head and neck cancer (HNC) is associated with substantial morbidity, functional impairment, and represents a substantial socioeconomic burden worldwide. Although advances in surgery, radiotherapy (RT), and systemic therapies have improved oncologic outcomes, treatment-related toxicities frequently overlap with profound economic consequences for patients and caregivers. Financial toxicity (FT), defined as the objective financial burden and subjective financial distress associated with cancer care, has emerged as a clinically relevant determinant of treatment adherence, quality of life (QoL), and survival. In HNC, FT appears particularly critical during active treatment, when multimodal therapies, nutritional compromise, work interruption, transportation costs, and supportive care needs converge. Methods: This structured narrative review synthesizes current literature regarding FT during active treatment for HNC, focusing on conceptual definitions, measurement approaches, temporal dynamics, clinical and psychosocial consequences, and health system determinants. The review was conducted according to the Scale for the Assessment of Narrative Review Articles (SANRA) framework using searches of PubMed/MEDLINE, Scopus, Embase, and Google Scholar. Results: Available evidence suggests that FT may emerge early during treatment and evolve dynamically throughout the care trajectory. Lower socioeconomic status, treatment intensity, work interruption, and limited social support have been reported in association with increased FT. FT has also been linked to worse health-related QoL, higher symptom burden, treatment interruptions, hospitalization, and reduced survival, although the limited number of studies and methodological heterogeneity preclude definitive conclusions. Emerging evidence suggests that FT may be partially modifiable through interventions such as financial counseling and patient navigation. Conclusions: Future research should prioritize prospective and interventional designs using standardized multidimensional instruments capable of capturing both objective and subjective domains of FT. Integrating routine FT assessment into supportive oncology workflows may help identify vulnerable patients and support more equitable, patient-centered HNC care.

## Simple Summary

Head and neck cancer (HNC) is associated with substantial morbidity, functional impairment, and socioeconomic burden. During active treatment, patients may experience treatment-related toxicities, nutritional compromise, work interruption, transportation costs, and increased supportive care needs, all of which can contribute to financial toxicity (FT). This structured narrative review summarizes current evidence on FT during active treatment for HNC, focusing on definitions, measurement approaches, temporal patterns, clinical and psychosocial consequences, and health system determinants. Available evidence suggests that FT may emerge early, evolve throughout treatment, and be associated with poorer quality of life, higher symptom burden, treatment interruptions, hospitalization, and adverse clinical outcomes. Routine assessment of FT may help identify vulnerable patients and support earlier counseling, patient navigation, and more equitable, patient-centered cancer care.

## 1. Introduction

Head and neck cancers (HNCs) collectively represent one of the most common malignancies worldwide, with approximately 900,000 new cases and 450,000 deaths annually [1]. Despite advances in surgery, radiotherapy (RT), chemotherapy, and multimodal treatment strategies, patients with HNC continue to experience substantial treatment-related morbidity, including dysphagia, oral toxicities, nutritional compromise, communication impairment, and psychosocial distress [2,3,4].

Current management of HNC is individualized according to tumor site, disease stage, resectability, histologic subtype, viral status when applicable, patient comorbidities, functional status, and multidisciplinary treatment planning. Early-stage tumors may be managed with single-modality treatment, such as surgery or RT, whereas locally advanced disease frequently requires multimodal therapy, including surgery followed by risk-adapted adjuvant RT or chemoradiotherapy (CRT), or definitive RT/CRT. In recurrent or metastatic settings, systemic treatment options may include platinum-based chemotherapy, cetuximab-based targeted therapy, and immune checkpoint inhibitors, such as pembrolizumab or nivolumab, in selected clinical contexts. Although these approaches have improved disease control for many patients, they also increase treatment complexity, supportive care needs, and the potential for financial toxicity (FT) [5,6,7].

Within this context, FT has emerged as an increasingly recognized determinant of health outcomes in oncology. FT encompasses both objective financial burdens, including out-of-pocket expenses and income loss, and subjective financial distress related to treatment-associated economic strain [8,9,10,11]. In HNC, FT assumes particular relevance because the period of greatest financial vulnerability frequently overlaps with the peak of treatment-related toxicity during active therapy.

Growing evidence suggests that FT is associated with reduced treatment adherence, impaired quality of life (QoL), increased symptom burden, and worse survival outcomes [8,12,13,14,15,16]. Nevertheless, despite its clinical importance, FT during active treatment for HNC remains insufficiently characterized.

Several studies on FT and financial hardship in HNC have primarily focused on survivorship, follow-up care, or post-treatment populations [14,15,17,18,19,20,21]. In contrast, among the studies identified in this review, only seven primary studies specifically addressed FT during active treatment or the peri-treatment period [22,23,24,25,26,27,28], indicating that evidence focused on the active treatment phase remains comparatively limited and methodologically heterogeneous.

HNC may be particularly susceptible to FT due to the convergence of several clinical, functional, and socioeconomic factors. Advanced-stage presentation is one contributor to this vulnerability, although its magnitude varies across populations and healthcare systems. In selected cohorts included in this review, advanced disease was frequently reported among patients undergoing active or peri-treatment assessment. For example, stage IV disease represented 57.7% of patients in a Canadian HNC cohort and 49% of patients in a United States cohort undergoing RT, whereas stage III or IV disease represented 78.7% of patients in a Chinese nasopharyngeal carcinoma cohort [24,26,27]. In a large Brazilian cohort comprising 3052 patients with head and neck squamous cell carcinoma, 87.9% presented with stage III or IV disease at diagnosis, including approximately 74% with stage IV tumors [7]. These data are not intended to indicate that the present review is primarily focused on Brazil, but rather to illustrate how advanced-stage presentation may increase treatment intensity, healthcare utilization, and supportive care needs in settings where late diagnosis remains frequent. Advanced-stage disease is associated with poorer survival outcomes, greater treatment complexity, and higher supportive care needs, all of which may contribute to financial burden throughout the cancer care continuum [7,29].

Furthermore, patients often navigate multiple healthcare services before receiving a definitive diagnosis and initiating treatment. Delays in diagnosis and fragmented referral pathways remain common and may increase healthcare utilization, transportation expenses, work absenteeism, productivity losses, and caregiver burden throughout the cancer journey [29].

Treatment-related toxicities are frequent among patients undergoing HNC treatment, particularly among those receiving RT or CRT. Acute adverse effects commonly include oral mucositis, dysphagia, odynophagia, xerostomia, dysgeusia, pain, dermatitis, fatigue, nutritional compromise, and speech impairment, whereas late complications may include persistent xerostomia, dysphagia, trismus, dental disease, osteoradionecrosis, fibrosis, and long-term speech or swallowing dysfunction [2,3,4]. These toxicities may impair oral intake, increase the need for analgesics, nutritional supplementation, feeding tube placement, emergency department visits, or hospitalization, and substantially affect patients’ ability to maintain employment or return to work [19,26,28]. Clinically, severe toxicities may also lead to treatment delays, unplanned treatment interruptions, dose modifications, or premature discontinuation, potentially compromising treatment intensity and overall effectiveness [12,26,28]. Consequently, treatment-related toxicities may exacerbate both direct and indirect financial burden during active treatment and survivorship [14,24].

Given the clinical relevance of this issue, this structured narrative review aims to critically synthesize the current evidence regarding FT during active treatment for HNC, focusing on conceptual definitions, measurement approaches, temporal trajectories, clinical and psychosocial associations, and health system determinants. Particular emphasis is placed on identifying methodological gaps, structural drivers of vulnerability, and future directions for supportive oncology and health policy research. Given the conceptual, methodological, and clinical heterogeneity of the available literature, a narrative approach was considered the most appropriate to integrate findings across diverse study designs and domains.

## 2. Methods

This narrative review was conducted in accordance with the Scale for the Assessment of Narrative Review Articles (SANRA) guidelines [30], with the aim of providing a structured and transparent synthesis of the current evidence on FT in HNC.

### 2.1. Search Strategy and Data Sources

A structured literature search was performed across multiple electronic databases, including PubMed/MEDLINE, Scopus, and Embase. Additional sources, such as Google Scholar and ProQuest, were also searched. The search encompassed articles published between January 2010 and February 2026, reflecting the emergence and consolidation of the concept of FT in oncology research.

The search strategy combined controlled vocabulary and free-text terms related to FT and HNC. Key terms included: “financial toxicity”, “financial burden”, “financial hardship”, “financial impact”, “healthcare costs”, “cost of treatment”, “cost of illness”, “out-of-pocket costs”, “economic burden”, “head and neck cancer”, “oral cancer”, and “oncologic treatment”. Boolean operators (AND/OR) were used to refine the search and optimize both sensitivity and specificity.

Reference lists of selected articles were manually screened to identify additional relevant studies not captured in the initial search. Eligible studies included those involving patients with HNC that examined FT or related economic outcomes and reported clinical, psychosocial, or economic endpoints, such as QoL, treatment adherence, or survival. Publications were restricted to those written in English, Portuguese, or Spanish.

Both quantitative and qualitative designs were considered, including observational studies (cross-sectional, cohort, and retrospective) and interventional studies, to reflect the multidimensional nature of FT, encompassing both objective financial measures and subjective patient-reported experiences. Systematic reviews, meta-analyses, editorials, commentaries, and conference abstracts were excluded to prioritize primary evidence and minimize duplication.

The selection of studies was guided by three predefined domains: population, outcome, and treatment phase. Studies were considered eligible when they included patients with HNC, evaluated FT or related economic outcomes, and reported data relevant to active treatment or treatment-associated clinical, psychosocial, or economic consequences. Records were initially screened by title and abstract, followed by full-text assessment when appropriate. Articles were excluded when they did not focus on HNC, did not evaluate FT or related economic outcomes, focused exclusively on survivorship or general healthcare costs, were secondary literature, or did not provide data relevant to active treatment. Uncertain cases were reassessed in relation to the review aim and predefined eligibility domains. Formal inter-rater reliability coefficients for study selection or thematic coding were not calculated. Study eligibility and thematic classification were instead guided by predefined eligibility domains, iterative reading of the selected studies, and alignment with the SANRA-based [30] structured narrative review framework. Eligible primary studies were incorporated into the thematic narrative synthesis and summarized in Table 1 when they specifically addressed FT during active treatment.

### 2.2. Data Extraction, Synthesis, and Analytical Framework

Data extraction was conducted through iterative reading and thematic organization of the selected studies. Information was systematically collected on study design, population characteristics, measures of FT, associated risk factors, and reported clinical and psychosocial outcomes.

Given the heterogeneity of study designs, populations, and outcome measures, a quantitative synthesis was not considered appropriate. Instead, findings were integrated using a narrative approach, with emphasis on identifying recurring patterns, conceptual frameworks, and associations reported across studies. Attention was given to the distinction between objective financial burden, such as out-of-pocket expenses and income loss, and subjective financial distress, reflecting patients that perceived economic strain and psychological responses to treatment-related costs.

To ensure a structured synthesis in accordance with SANRA principles [30], the evidence was organized into interconnected thematic domains, including conceptualization of FT, determinants and risk factors, clinical and psychosocial consequences, temporal dynamics during active treatment, caregiver and household burden, measurement approaches, and mitigation strategies.

## 3. Literature Review

### 3.1. Conceptualizing Financial Toxicity in Head and Neck Cancer

FT represents a multidimensional construct encompassing both objective financial burden and subjective financial distress associated with cancer care [8,9,11]. Direct costs include transportation, lodging, copayments, supportive medications, nutritional supplementation, dental care, and out-of-network consultations, whereas indirect costs arise from work interruption, productivity loss, unemployment, early retirement, and long-term reductions in earning capacity [8,16,31].

Objective financial burden should be interpreted within the broader context of healthcare policy, insurance coverage, reimbursement structures, and social protection mechanisms, which strongly influence out-of-pocket expenditures, income replacement, transportation support, and access to supportive care resources [8,24,31]. Across health systems, the magnitude and consequences of FT may therefore vary substantially according to public coverage, social welfare protections, and the extent to which indirect and supportive care costs are reimbursed [22,23,24]. Accordingly, this review does not seek to make causal comparisons across countries or healthcare systems. Rather, it considers how HNC-specific clinical and functional vulnerabilities may interact with health-system and social-policy contexts to shape financial hardship during active treatment [2,3,4,26].

Importantly, contemporary conceptual frameworks recognize FT as a multidimensional phenomenon that extends beyond treatment-related expenses alone. In addition to objective financial burden and subjective financial distress, FT encompasses the broader interaction between economic hardship, psychosocial well-being, and healthcare experiences throughout the cancer continuum. This perspective acknowledges that financial challenges may influence patients’ QoL, access to supportive care, treatment experiences, and overall engagement with healthcare services, reinforcing the relevance of FT as a patient-centered outcome in oncology [8,10,11].

Within specific healthcare and social-policy contexts, patients with HNC may be particularly vulnerable to FT because treatment-related toxicities frequently impair speech, swallowing, nutrition, communication, and social participation, thereby amplifying functional disability, caregiver dependence, and limitations in workforce participation [2,3,4,25,26,28].

The burden of FT extends beyond patients themselves and often affects caregivers and household economic stability. Recurrent treatment-related expenses, prolonged clinic attendance, and simultaneous productivity loss among caregivers may precipitate substantial psychosocial strain and long-term economic consequences for families [8,16,25].

Caregiver-related financial burden has gained increasing recognition as an integral component of FT. In addition to direct caregiving responsibilities, family members frequently experience work absenteeism, reduced productivity, income loss, and increased out-of-pocket expenditures related to transportation, accommodation, and supportive care. Emerging evidence suggests that financial hardship experienced by caregivers may contribute to household economic instability and psychosocial distress, reinforcing the concept that FT should be viewed as a family-level rather than exclusively patient-level phenomenon [16,25].

### 3.2. Structural and Socioeconomic Determinants of Financial Toxicity

The development and severity of FT are strongly influenced by structural inequities and healthcare system characteristics. HNC disproportionately affects socially vulnerable populations, including individuals with lower educational attainment, unstable employment, reduced health literacy, and limited financial reserves [6,32,33]. These social determinants reduce financial resilience during prolonged oncologic treatment.

Recent studies have further highlighted the multifactorial nature of FT in HNC. In a prospective cohort of patients undergoing radiation therapy, Harada et al. (2025) [26] identified younger age, unemployment, Medicaid insurance coverage, single marital status, advanced T-stage disease, and concurrent CRT as significant factors associated with worse FT scores. Importantly, these socioeconomic vulnerabilities frequently coexisted with markers of greater clinical complexity and treatment burden, suggesting that financial hardship often emerges from the intersection of social disadvantage and disease severity. Similarly, Luo et al. (2025) [27] reported that lower monthly income, less favorable coping strategies, and reduced social support were independently associated with higher levels of FT among patients with nasopharyngeal carcinoma, reinforcing the importance of both socioeconomic and psychosocial determinants in shaping financial outcomes during cancer treatment.

In many countries, particularly low- and middle-income settings, fragmented insurance systems, high out-of-pocket expenditures, and limited social protection mechanisms increase the risk of catastrophic healthcare spending and treatment discontinuation [6]. Nevertheless, FT is not restricted to resource-limited environments. Evidence from the United States demonstrates that patients remain vulnerable to copayments, transportation costs, income loss, and uncovered ancillary expenses despite public insurance programs such as Medicare and Medicaid [12,13,19,20,34,35]. These observations suggest that FT should be interpreted as a structural challenge whose magnitude and clinical consequences vary according to healthcare coverage, reimbursement policies, social protection mechanisms, and patient-level vulnerability, rather than as a phenomenon restricted to specific geographic or economic contexts.

These findings collectively indicate that FT results from a complex interaction between patient-level vulnerabilities, disease-related factors, treatment intensity, psychosocial resources, and health-system characteristics, rather than from financial resources alone [26,27].

### 3.3. Measurement Approaches and Methodological Variability

Assessment of FT during active treatment varies substantially across studies. Egestad et al. (2015) [22] and Farrugia et al. (2021) [23] assessed financial difficulty using a single item from the EORTC QLQ-C30 (question 28), allowing pragmatic integration into broader quality-of-life instruments but providing limited evaluation of indirect costs and subjective financial distress.

More recent studies have increasingly adopted multidimensional instruments such as the COmprehensive Score for Financial Toxicity (COST). Nguyen et al. (2023) [25], Harada et al. (2025) [26], Luo et al. (2025) [27], and Mady et al. (2025) [28] employed COST-based approaches to evaluate patient-reported financial distress across multiple domains.

The adoption of multidimensional instruments has contributed substantially to a more comprehensive understanding of FT in HNC. Using the COST instrument, Nguyen et al. (2023) [25] demonstrated that both patients and caregivers experience considerable levels of financial distress, with caregivers in some cases reporting greater financial concerns than patients themselves. Similarly, Harada et al. (2025) [26] found that approximately 42% of patients undergoing RT exhibited clinically significant FT, which was associated with poorer health-related QoL, increased symptom burden, higher rates of emergency department visits and hospitalizations, and inferior overall survival. These findings illustrate the value of multidimensional assessment tools in capturing the broader clinical and psychosocial implications of FT beyond direct economic burden alone.

Khan et al. (2022) [24] adopted a complementary perspective by focusing on objective out-of-pocket expenditures related to travel, accommodation, meals, medical costs, and ancillary services. Together, these approaches illustrate the conceptual heterogeneity currently characterizing FT research in HNC. Importantly, methodological variability remains substantial across studies, including differences in study design, timing of assessment, healthcare systems, and outcome definitions. This heterogeneity currently limits direct comparisons and complicates the development of standardized evidence-informed interventions.

An additional methodological challenge relates to the predominantly cross-sectional or episodic assessment of FT. Most available instruments capture financial burden at isolated time points and may not adequately reflect the dynamic fluctuations in financial hardship that occur throughout active treatment and recovery. Emerging digital health technologies, including mobile applications, electronic patient-reported outcome platforms, and remote monitoring systems, may offer novel opportunities for longitudinal assessment of FT by enabling real-time collection of patient-reported financial data and healthcare-related expenditures. Although these approaches remain largely unexplored in HNC, they may help improve measurement precision and facilitate earlier identification of financially vulnerable patients [8,31].

The increasing use of validated multidimensional instruments represents an important step toward improving comparability across studies and facilitating the development of targeted interventions and supportive care strategies for patients at greatest risk of FT.

### 3.4. Financial Toxicity During Active Treatment

Evidence specifically addressing FT during active treatment for HNC remains comparatively limited. Most available studies have traditionally focused on survivorship populations, particularly in high-income settings [12,13,19,20,34,35]. The active treatment period represents a particularly vulnerable phase because multimodal therapies frequently coincide with severe symptom burden, nutritional compromise, work interruption, and repeated healthcare utilization. Available studies suggest that FT may already be present at treatment initiation and continues to evolve dynamically throughout treatment. The main characteristics of studies evaluating FT during active treatment in patients with HNC are summarized in Table 1.

Overall, seven primary studies specifically addressing FT during active treatment or the peri-treatment period were identified and summarized [22,23,24,25,26,27,28]. These studies were published between 2015 and 2025 and were conducted in Norway, the United States, Canada, and China. Study designs included prospective observational studies, retrospective analyses of prospectively collected data, cross-sectional studies, and prospective mixed-methods cohorts. The timing of FT assessment varied across studies, including treatment initiation, the first and last weeks of RT, mid-treatment or post-surgical assessments, and early post-treatment follow-up. This variation in geographic setting, study design, treatment phase, and measurement approach underscores both the emerging nature of this evidence base and the current methodological heterogeneity of FT research in HNC.

Because objective financial burden is a central component of FT, the cost-related factors described across the included studies can be organized into direct and indirect cost dimensions, including treatment-related out-of-pocket expenses, supportive care expenditures, income loss, productivity loss, caregiver time, and household-level economic burden, as summarized in Table 2.

The temporal evolution of FT during active treatment likely reflects the cumulative impact of treatment-related toxicity, healthcare utilization, and disruption of employment and daily functioning. Khan et al. (2022) [24] demonstrated that out-of-pocket expenditures were highest during active treatment, particularly among patients receiving multimodal therapies, and gradually declined during follow-up. Similarly, longitudinal analyses by Mady et al. (2025) [28] showed that financial hardship may persist beyond treatment completion, although trajectories vary according to socioeconomic status and baseline vulnerability. These findings suggest that FT should be viewed as a dynamic process that evolves throughout treatment and survivorship rather than as a static condition measured at a single time point [24,26,28].

The timing of FT assessment may therefore substantially influence reported prevalence estimates and observed associations with clinical outcomes. Cross-sectional evaluations performed at treatment initiation may underestimate the cumulative economic burden experienced later in therapy, whereas post-treatment assessments may fail to capture periods of acute financial vulnerability. These considerations highlight the importance of longitudinal monitoring approaches capable of capturing fluctuations in financial burden across different treatment phases and recovery periods [24,28,31].

Nguyen et al. (2023) [25], in a cross-sectional pilot study involving patients at treatment initiation, identified measurable financial burden early in the treatment course using the COST instrument. Egestad et al. (2015) [22] observed relatively stable financial difficulty scores during RT in Norway, whereas Farrugia et al. (2021) [23] demonstrated worsening financial difficulty among patients not receiving financial counseling during RT in the United States.

Longitudinal evidence further supports the dynamic nature of FT during treatment. Khan et al. (2022) [24] demonstrated that out-of-pocket costs peaked during active therapy, particularly among patients receiving CRT or surgery followed by adjuvant treatment and progressively declined over time. Similarly, Harada et al. (2025) [26] and Mady et al. (2025) [28] demonstrated that FT trajectories are influenced by socioeconomic status, treatment intensity, and recovery phase.

### 3.5. Clinical and Psychosocial Consequences

Growing evidence demonstrates that FT is associated with clinically relevant adverse outcomes in HNC. Harada et al. (2025) [26] reported that lower COST scores were significantly associated with worse health-related QoL, increased treatment-related morbidity, hospitalization, feeding tube placement, and reduced overall survival.

Similarly, Mady et al. (2025) [28] demonstrated persistent associations between FT and reduced QoL over time, even after partial post-treatment recovery. Luo et al. (2025) [27] further identified significant associations between FT, coping strategies, perceived social support, and psychosocial distress among patients with nasopharyngeal carcinoma.

Importantly, the consequences of FT extend beyond patient-reported outcomes and may manifest as measurable clinical events. In the prospective study by Harada et al. (2025) [26], patients with worse FT experienced significantly higher rates of emergency department visits, hospitalizations, and feeding tube placement during treatment. These findings suggest that FT may function not only as a marker of economic hardship but also as an indicator of broader clinical vulnerability, potentially reflecting the complex interplay between socioeconomic disadvantage, disease severity, symptom burden, and treatment-related complications [26].

The relationship between FT and clinical outcomes is likely bidirectional. While financial hardship may contribute to treatment delays, reduced adherence, and barriers to supportive care, patients with more advanced disease, greater symptom burden, and higher healthcare utilization may simultaneously experience increased financial strain. This reciprocal interaction suggests that FT should be considered both a consequence of cancer-related morbidity and a potential contributor to worsening health outcomes, further supporting its inclusion as a routinely assessed domain of comprehensive cancer care [8,26].

Beyond psychosocial outcomes, FT appears to be associated with treatment delays, reduced adherence, and premature discontinuation of therapy [8,12]. In patients with HNC, treatment-related functional limitations, including dysphagia, speech impairment, nutritional compromise, and disfigurement, may reduce QoL, social participation, and ability to work [2,3,4]. Financial hardship may further compound these challenges through associations with psychosocial distress, reduced treatment adherence, and barriers to supportive care, although the extent of material hardship is strongly modified by healthcare coverage, reimbursement policies, income protection, and access to social support services [8,24,26,28].

### 3.6. Health System Context and Opportunities for Intervention

Objective financial burden is strongly mediated by healthcare policy, reimbursement structures, and social protection systems; therefore, cross-country comparisons of FT should be interpreted cautiously [8,24,31]. In this review, the available studies are interpreted as illustrating how different health-system contexts may shape the experience and consequences of FT, rather than as providing causal comparisons between countries. Egestad et al. (2015) [22], conducted in Norway, demonstrated relatively low and stable financial difficulty scores, consistent with strong public healthcare coverage and social welfare support. In contrast, Farrugia et al. (2021) [23] demonstrated increased financial difficulty during RT among patients not receiving financial counseling in the United States. Of particular relevance, financial counseling appeared associated with stabilization of financial burden, suggesting that FT may be partially modifiable through supportive interventions.

Emerging evidence suggests that effective mitigation of FT will likely require interventions operating at multiple levels of care. At the patient level, financial counseling, social work support, and patient navigation programs may facilitate access to available resources and reduce barriers to treatment adherence. At the institutional level, routine screening for FT using validated instruments may enable earlier identification of vulnerable patients and timely referral to supportive services. At the policy level, strategies aimed at reducing out-of-pocket expenditures, improving access to transportation and supportive care resources, and strengthening social protection mechanisms may further alleviate the economic burden associated with cancer treatment [8,23,26].

Despite growing recognition of FT, evidence supporting specific mitigation strategies remains limited. Most available interventions have focused on financial counseling and patient navigation, with relatively few studies evaluating their long-term effects on financial outcomes, QoL, healthcare utilization, or treatment adherence. Furthermore, the effectiveness of these interventions may vary substantially across healthcare systems, insurance models, and socioeconomic contexts. Future intervention studies should therefore incorporate standardized FT measures, longitudinal follow-up, and implementation outcomes to determine which strategies are most effective, scalable, and sustainable across diverse oncology settings [8,23,26].

Khan et al. (2022) [24] further demonstrated that substantial out-of-pocket costs persist even in universal healthcare systems because many supportive and indirect expenses remain insufficiently covered. These findings support incorporation of routine FT screening, financial counseling, patient navigation, and multidisciplinary supportive care into oncology workflows. Validated instruments such as COST may facilitate systematic identification of economically vulnerable patients and enable earlier supportive interventions.

Early identification of FT may be particularly important in HNC because many determinants of financial vulnerability are potentially recognizable before treatment initiation. Factors such as employment status, insurance coverage, socioeconomic disadvantage, transportation requirements, caregiver availability, and anticipated treatment intensity may help identify patients at increased risk of financial hardship. Incorporating routine FT screening into multidisciplinary pretreatment assessments could facilitate proactive referral to financial counseling, social work services, patient navigation programs, nutritional support, and community-based resources. Such strategies are consistent with contemporary models of supportive oncology that emphasize early recognition of modifiable risk factors and personalized supportive care throughout the cancer continuum. Importantly, timely interventions may improve not only financial outcomes but also treatment adherence, healthcare engagement, and overall patient experience [8,23,26].

### 3.7. Future Directions and Research Priorities

Current evidence regarding FT during active treatment for HNC remains limited by substantial methodological heterogeneity, small sample sizes, inconsistent measurement approaches, and predominance of studies conducted in high-income countries. Future research should prioritize prospective multicenter investigations using standardized multidimensional instruments capable of capturing both objective financial burden and subjective financial distress. Interventional studies evaluating financial counseling, patient navigation, transportation assistance, social prescription, social support interventions, and supportive care integration are also critically needed.

Emerging digital health technologies may represent an important avenue for advancing FT research and clinical management. Mobile applications, electronic patient-reported outcome platforms, and remote monitoring systems could enable real-time collection of direct and indirect cost data, facilitate early identification of financially vulnerable patients, and support timely referral to financial counseling and supportive services. Such approaches may be particularly valuable in HNC, where treatment-related toxicities, frequent healthcare encounters, and rapid changes in functional status can substantially influence financial burden throughout treatment [8,26].

Additional research should address structurally vulnerable populations, including patients from low- and middle-income countries and historically marginalized groups within high-income settings [32,33]. Additional methodological research is needed to develop and validate innovative approaches capable of capturing the dynamic evolution of FT throughout the cancer continuum. Prospective monitoring of out-of-pocket expenditures, productivity losses, caregiver burden, and treatment-related costs through digital platforms may provide a more comprehensive understanding of financial hardship than currently available cross-sectional assessments and retrospective surveys [8,31]. Implementation science and real-world data may further contribute to the development of scalable and context-sensitive mitigation strategies.

Future studies should also prioritize implementation science approaches aimed at integrating FT screening into routine oncology workflows. Evaluating the feasibility, acceptability, cost-effectiveness, and sustainability of FT mitigation strategies across different healthcare systems will be essential for translating research findings into clinical practice. Attention should be given to interventions that can be scaled across diverse healthcare settings while maintaining sensitivity to local socioeconomic realities and resource constraints [3,8,33].

## 4. Conclusions

FT during active treatment for HNC appears clinically relevant but remains incompletely characterized. Available evidence suggests that financial burden may emerge early during treatment and may be associated with impaired QoL, increased morbidity, reduced treatment adherence, healthcare utilization, and adverse clinical outcomes. However, the limited number of studies and substantial heterogeneity in healthcare context, study design, populations, timing of assessment, and FT measures preclude definitive conclusions and limit direct comparisons across studies.

Importantly, FT should not be interpreted solely as an economic consequence of cancer treatment, but rather as a multidimensional and potentially modifiable determinant of health shaped by healthcare systems, social protection structures, reimbursement policies, and supportive care access. Integrating routine FT assessment into supportive oncology workflows may help identify financially vulnerable patients earlier and support more patient-centered care. Future research should prioritize standardized measurement approaches and prospective interventional studies capable of clarifying the temporal trajectory of FT and informing evidence-based mitigation strategies in HNC care.

Emerging digital health technologies, including remote monitoring platforms and electronic patient-reported outcome systems, may offer opportunities for improving longitudinal assessment and early detection of FT throughout the treatment trajectory. Furthermore, implementation science approaches will be essential to translate emerging evidence into routine oncology practice and to develop scalable, context-sensitive interventions capable of reducing financial hardship across diverse healthcare settings. Addressing FT effectively will require coordinated efforts spanning patients, healthcare institutions, policymakers, and health systems to promote more equitable and patient-centered cancer care.

## Figures and Tables

**Table 1:** Characteristics of studies evaluating FT during active treatment in patients with HNC.

Author/Year	Country	Study Design	Tumor Type	Patients (*n*)	Sex Distribution	Tumor Localization	Intervention/Instrument	Timing of Assessment
Egestad et al. (2015) [22]	Norway	Prospective observational	HNC	67	Male 49 (73.1%); Female 18 (26.9%)	Oral cavity 17 (25.4%); pharynx 16 (23.9%); larynx 16 (23.9%); salivary glands 7 (10.4%); other/unknown 11 (16.4%)	EORTC QLQ-C30 [single-item financial difficulty (Q28) with four response categories (“not at all,” “a little,” “quite a bit,” or “very much”)]	During RT (first and last week of RT)
Farrugia et al. (2021) [23]	United States	Retrospective analysis of prospectively collected data	HNC	387	Male 302 (78.0%); Female 85 (22.0%)	Pharynx 204 (52.7%); non-pharynx 183 (47.3%)	EORTC QLQ-C30 [single-item financial difficulty, (Q28) with four response categories (“not at all,” “a little,” “quite a bit,” or “very much”)]	During RT (first and last week of RT)
Khan et al. (2022) [24]	Canada	Prospective observational	HNC	657	Male 508 (77.3%); Female 149 (22.7%)	Oropharynx 304 (46.3%); lip/oral cavity 144 (21.9%); larynx 99 (15.1%); nasopharynx 42 (6.4%); hypopharynx 19 (2.9%); nasal cavity 11 (1.7%); other/unknown primary 38 (5.8%)	Out-of-pocket cost questionnaire (self-reported—including travel, accommodation, medical and ancillary costs)	During treatment (mid-RT and post-surgery) and post-treatment (3, 6, 12, and 24 months)
Nguyen et al. (2023) [25]	United States	Cross-sectional pilot study	HNC	27	Male 17 (63.0%); Female 10 (37.0%)	Laryngeal 1 (3.7%); nasopharyngeal 2 (7.4%); oral cavity 7 (25.9%); oropharyngeal 12 (44.4%); other 5 (18.5%)	COST	At treatment initiation (single time point)
Harada et al. (2025) [26]	United States	Prospective observational	HNC	74	Male 54 (73.0%); Female 20 (27.0%)	Oral cavity 18 (24%); oropharynx 3 (4%); larynx/hypopharynx 6 (8%); nasopharynx 3 (4%); sinonasal 6 (8%); salivary gland 3 (4%); thyroid 3 (4%); orbit 3 (4%); cutaneous 25 (34%); neck 4 (5%)	COST (FACIT-COST, 11-item validated instrument)	Pre-RT and post-RT (2–12 weeks after RT)
Luo et al. (2025) [27]	China	Cross-sectional study	HNC	155	Male 126 (81.3%); Female 29 (18.7%)	Nasopharynx 155 (100%)	COST (COST-PROM), MCMQ, PSSS	During RT (assessment based on previous 7 days)
Mady et al. (2025) [28]	United States	Prospective cohort (mixed methods)	HNC	64	Male 52 (81%); Female 12 (19%)	Oral cavity 16 (25%); oropharynx 27 (42%); larynx/hypopharynx 13 (20%); other 8 (13%)	COST (v1), FDQ, UW-QOL	At diagnosis, 3 and 6 months post-diagnosis (longitudinal)

**Table 2:** Direct and indirect cost-related factors contributing to financial toxicity during active treatment for head and neck cancer.

Cost Dimension	Subcategory	Cost-Related Factors Addressed in the Review
**Direct costs**	Transportation and travel-related expenses	Transportation to treatment centers; repeated travel for radiotherapy, chemotherapy, surgery, supportive care visits, or follow-up appointments
	Lodging and meal-related expenses	Accommodation near treatment centers; meals during prolonged treatment days; expenses related to geographic distance from oncology services
	Medical and treatment-related expenses	Copayments; out-of-pocket medical costs; out-of-network consultations; ancillary medical costs
	Supportive medication and symptom-management expenses	Analgesics; topical agents; medications for treatment-related toxicities; supportive prescriptions required during active therapy
	Nutritional support expenses	Nutritional supplements; dietary modifications; feeding-related supplies; supportive nutritional care during treatment
	Dental and oral supportive care expenses	Pretreatment dental evaluation; radiographs; restorative and periodontal care; indicated extractions before radiotherapy; fluoride-based preventive care; management of oral mucositis, dental infections, xerostomia, radiation-related caries, trismus, and long-term oral complications.
**Indirect costs**	Employment disruption	Work interruption; work absenteeism; inability to maintain employment during active treatment; unemployment
	Productivity loss	Reduced productivity; decreased work capacity; limitations in daily functioning due to treatment-related morbidity
	Income loss and reduced earning capacity	Loss of income; reduced household earnings; early retirement; long-term reduction in earning capacity
	Caregiver-related productivity loss	Caregiver absenteeism; reduced caregiver productivity; caregiver time dedicated to transportation, appointments, and supportive care
	Household-level economic burden	Family-level financial strain; household income disruption; cumulative economic impact on patients and caregivers
	Time-related work and productivity loss	Work or productive time lost by patients or caregivers due to repeated clinic visits, waiting time, emergency department visits, hospitalizations, supportive care encounters, and treatment-related appointments.

## Data Availability

No new data were created or analyzed in this study. Data sharing is not applicable to this article.
